# Cincinnati Medical Society

**Published:** 1836

**Authors:** 


### Art. VI.—Cincinnati Medical Society.

The existence and objects of this Institution has been noticed from time to time in our Journal. It holds its meetings on Wednesday evening of each week, for seven months in the year, commencing with the 1st week of October. Its officers are elected on the fourth of March annually. During the winter the debates have been animated and useful to all of the members, but especially to students and Juniors in the profession. Its officers for the present year are: L. C. Rives, M. D., President, J. M. Mason, M. D., and S. D. Gross, M. D., Vice Presidents, Wm. Wood, M. D., Chairman, J. L. Riddell, M. D., Treasurer, and Librarian, C. A. Trimble, M. D., Secretary. J. P. Harrison, M. D., was chosen to deliver an annual address about the first of January next. W. W.

